# Unifying the Synthesis of a Whole Family of Marine Meroterpenoids through a Biosynthetically Inspired Sequence of 1,2-Hydride and Methyl Shifts as Key Step

**DOI:** 10.3390/md21020118

**Published:** 2023-02-10

**Authors:** Antonio Rosales Martínez, Román Nicolay Rodríguez-Maecker, Ignacio Rodríguez-García

**Affiliations:** 1 Department of Chemical Engineering, Escuela Politécnica Superior, University of Sevilla, 41011 Sevilla, Spain; 2 Department of Energy and Mechanics, Carrera de Ingeniería Petroquímica, Universidad de las Fuerzas Armadas-ESPE, Latacunga 050150, Ecuador; 3 Organic Chemistry, ceiA3, CIAIMBITAL, University of Almería, 04120 Almería, Spain

**Keywords:** marine natural products, meroterpenoids, rearrangements, synthetic strategies

## Abstract

Marine meroterpenoids have attracted a great deal of attention from synthetic research groups due to their attractive and varied biological activities and their unique and diverse structures. In most cases, however, further biological studies have been severely limited mainly to the scarcity of natural supply and because almost none of the reported syntheses methods has enabled unified access for a large number of marine meroterpenoids with aureane and avarane skeletons. Based on our previous publications and the study of recent manuscripts on marine meroterpenoids, we have conceived a unified strategy for these fascinating marine compounds with aureane or avarane skeletons using available drimane compounds as starting materials. The key step is a biosynthetic sequence of 1,2-hydride and methyl shifts. This strategy is of great synthetic value to access marine meroterpenoids through easy chemical synthetic procedures. Finally, several retrosynthetic proposals are made for the future synthesis of several members of this class of meroterpenoids, focused on consolidating these 1,2-rearrangements as a versatile and unified strategy that could be widely used in the preparation of these marine meroterpenoids.

## 1. Introduction

**Figure 1:**
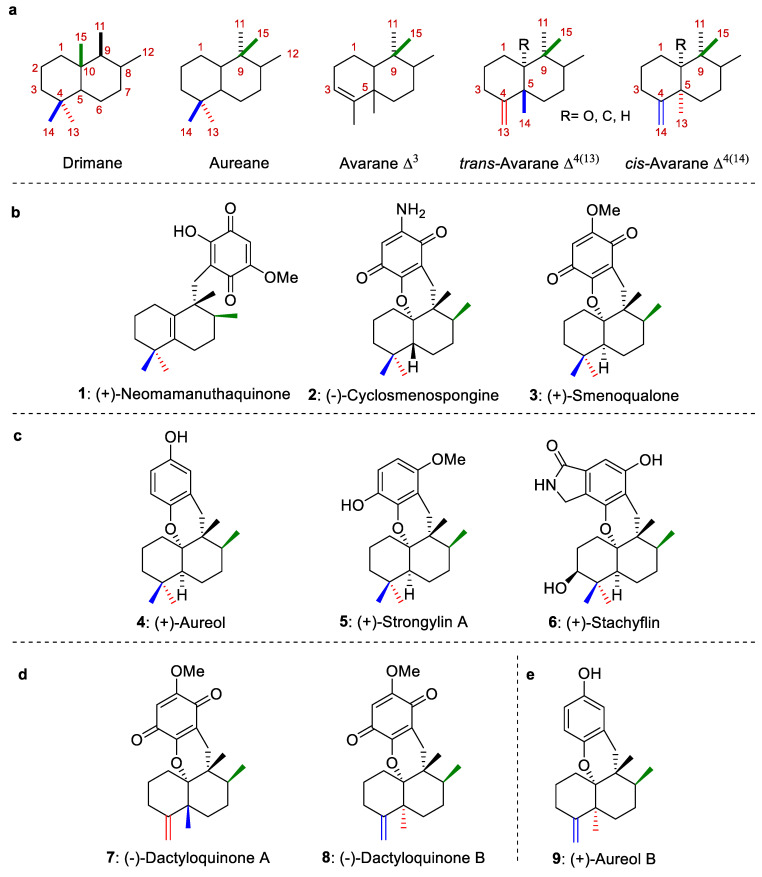
(**a**): Sesquiterpene skeletons. (**b**): Representative examples of sesquiterpene structures with aureane (quinone) skeletons. (**c**): Representative examples of sesquiterpene structures with aureane (hydroquinone) skeletons. (**d**): Representative examples of sesquiterpene structures with avarane (quinone) skeletons. (**e**): Representative examples of sesquiterpene structures with avarane (hydroquinone) skeletons.

Most marine sesquiterpene hydroquinones/quinones, which have been isolated from marine organisms, particularly from sponges and algae, possess an aureane skeleton or an avarane skeleton (Figure 1a), [1,2,3,4]. Biosynthetically, these avarane and aureane skeletons arise from 1,2-rearrangements of the drimane skeleton [2] (Figure 1a). In turn, natural compounds with avarane skeletons have been divided into two groups depending on their configuration at the C-5 position. The first one groups the avaranes, Δ^3^, while the second one includes the *trans*-avarane Δ^4(13)^ (*trans*-decalin system) or *cis-*avarane Δ^4(14)^ (*cis*-decalin system) (Figure 1a). Representative marine sesquiterpene structures with aureane and avarane skeletons are depicted in Figure 1b–e.

Many marine sesquiterpene quinones/hydroquinones exhibit a wide array of very interesting pharmacological properties, such as antiviral, cytotoxic, antimicrobial, antiinflamatory, antifungal and immunomodulation activities [2]. However, on many occasions, the study of the biological properties of these compounds has been limited due to the scarcity of marine natural supplies. This limitation is also due to the fact that the synthetic methodologies developed for these compounds are limited to individual compounds [5,6,7,8,9,10,11,12,13,14], and only the one described by Magauer and co-workers [15] allows access to a whole family of these fascinating marine natural products. Therefore, the development of an efficient strategy for the scalable construction of complex marine meroterpenoids with aureane and avarane skeletons is desirable from the viewpoint of pharmaceuticals, drug discovery, and medicinal chemistry.

**Scheme 1:**
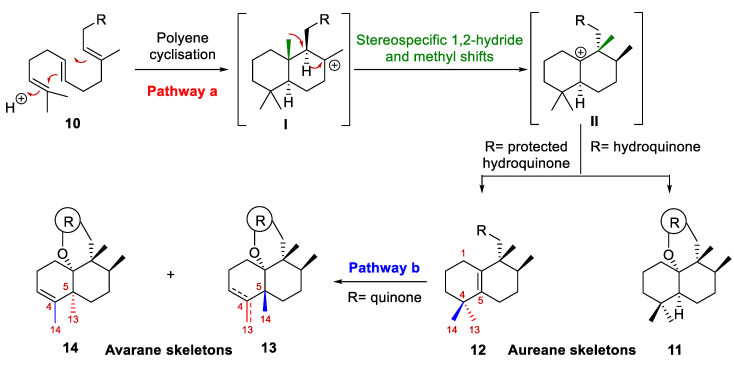
Proposed biosynthesis of the aureane skeleton (**10**, **11** and **12**). Progress in research on the synthesis of an avarane skeleton from an aureane skeleton (**12**, **13** and **14**).

Regarding their biogenesis [10,16,17,18,19], it was postulated that aureane skeleton formation presumably involves the diastereoselective cyclization of a polyene **10** to generate the tertiary carbocation **I**, which then would undergo a sequence of stereospecific 1,2-hydride and methyl shifts to give a new carbocation **II** (pathway a, Scheme 1). Depending on the nature of the R group, carbocation **II** could evolve into a tetrasubstituted alkene (**12**) or could undergo intramolecular attack by a hydroxy group present in the substituent R (usually an aromatic ring) to form a polycyclic system (**11**). Based on this biosynthetic proposal, different research groups have successfully synthesized different marine meroterpenoids with an aureane skeleton **11**–**12** [10,12,14,20,21]. Recently, the preparation of compounds with an avarane skeleton **13**–**14** has been reported using a quinone derivative of compound **12** through a cyclization reaction catalyzed by a Lewis acid, which causes the migration of the methyl groups, leading to two different configurations at the C-5 carbon (pathway b, Scheme 1) [14].

In this context, rearrangement reactions [22,23,24,25] offer a versatile way to generate the target structures through the bond reorganization of the intermediate precursors. Rearrangement reactions allow the straightforward formation of the core skeletons, the control of stereochemistry, and the identification of potential commercially available synthons.

**Scheme 2:**
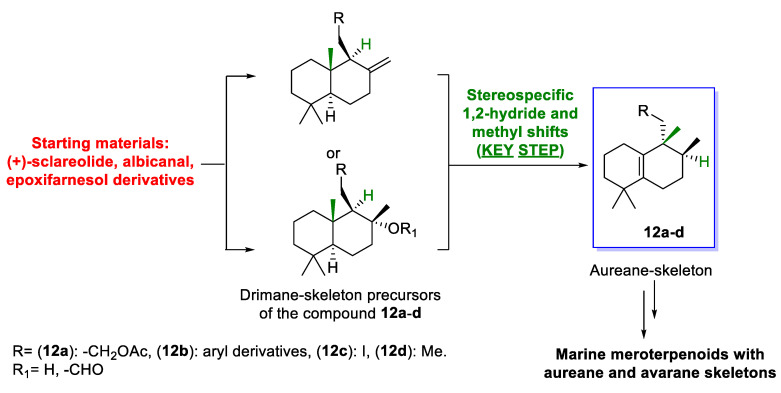
Conceptual model for the unified synthesis of marine meroterpenoids with aureane and avarane skeletons using 1,2-rearrangements as key step.

This article is focused on the synthetic efforts toward the compounds with aureane skeletons **12a**–**d** (Scheme 2). These compounds are formed from drimanes prepared from available starting material. The process involves a sequence of 1,2-hydride and methyl shifts as keys. Compounds **12a**–**d** are valuable intermediates for the synthesis of marine meroterpenoids with aureane or avarane skeletons, such as **1**–**9**. The above-mentioned key step can be considered a versatile, efficient, and promising unified methodology for the synthesis of meroterpenoids with aureane or avarane skeletons, including the control of the geometry of the stereocenters, the oxidative states, and the retrosynthetic relationships. In this way, compounds **12a**–**d**, as will be mentioned later, can be prepared through a short route from available drimanes, a process that is desirable considering the concept of step economy.

## 2. Synthesis of the Tetracyclic Alkenes 12a–d

### 2.1. George’s Synthesis of Compound ***12a***

**Scheme 3:**
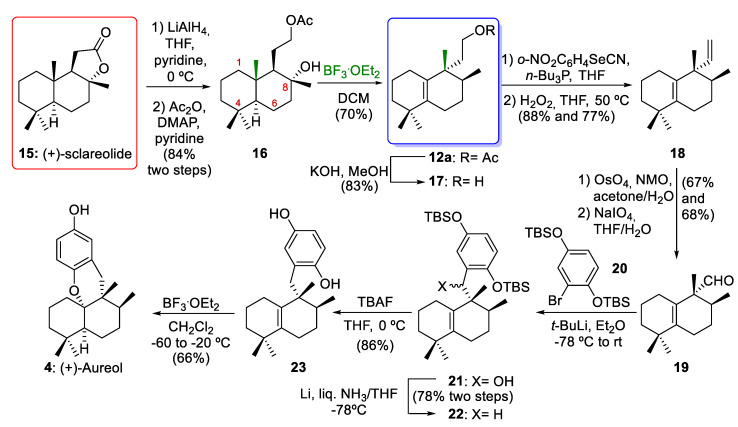
George´s synthesis of intermediate **12a** and total synthesis of (+)-aureol (**4**). BF_3_·Et_2_O = boron trifluoride etherate; DMAP = 4-(*N,N-*dimethylamino)pyridine; NM O = *N*-methylmorpholine-*N*-oxide; TBAF = tetrabutylammonium fluoride.

In 2012, George´s group [10] reported the enantioselective synthesis of **12a** via a biosynthetically inspired stereospecific sequence of 1,2-hydride and methyl shifts of **16** (Scheme 3). The reduction of commercially available (+)-sclareolide (**15**) afforded a diol. The selective acetylation of diol using Ac_2_O in pyridine afforded the acetate ester **16** (84% yield over two steps). Aureane compound **12a** arose from 1,2-rearrangements of drimane compound **16** using BF_3_·Et_2_O as a Lewis acid (70% yield). This key rearrangement proceeded via stereospecific 1,2-hydride and methyl shifts and yielded the desired compound **12a** in a good yield. As will be discussed in Section 3, this rearrangement occurs through the formation of a tertiary carbocation at C-8, which can then undergo a sequence of stereospecific 1,2-hydrogen and methyl shifts. With the aureane compound **12a** in their hands, George´s group [10] carried out the synthesis of the marine natural product (+)-aureol (**4**). First, alcohol **17** was prepared by the hydrolysis of acetate **12a** (83% yield). The excision of one carbon atom with a Grieco–Sharpless elimination followed by oxidative cleavage gave **19**. The resulting dialcohol underwent oxidative cleavage with NaIO_4_, leading to the aldehyde **19** (46% yield, two steps). Coupling aldehyde **19** with the aryllithium derivative of **20** formed a mixture of diastereomeric benzylic alcohols **21**. The reduction of compound **21** gave the deoxygenated compound **22** (78% yield over two steps). After treatment of compound **22** with TBAF hydroquinone, **23** was synthesized (86% yield). Finally, the reaction of compound **23** with BF_3_·Et_2_O gave (+)-aureol (**4**) with a yield of 66%. This final cyclization to obtain the marine tetracyclic meroterpenoid aureol (**4**) had been previously described by Marcos and co-workers [8] in their synthesis of (−)-aureol. In conclusion, the aureane intermediate **12a** was obtained through 1,2-rearrangements of the drimane compound **16** using BF_3_·Et_2_O as key step. This intermediate **12a** was the key compound for the total synthesis of (+)-aureol (**4**). Compound **4** was first isolated from a marine sponge [26] and possesses potent biological activities, including anti-influenza A virus activity [27], selective cytotoxicity against human tumour cells [28], antibiotic activity [15], and the ability to inhibit SARS-CoV-2 [29].

### 2.2. Rosales Martínez’s Synthesis of Compound ***12b***

**Scheme 4:**
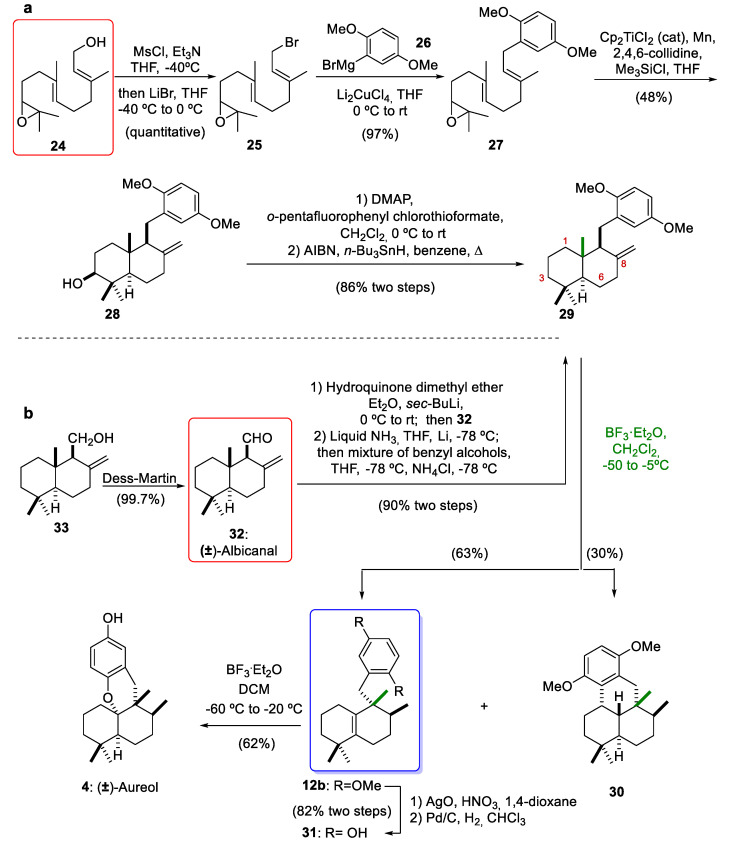
Rosales Martínez´s synthesis of compound **12b** by (**a**): Cp_2_TiCl-catalyzed radical cyclization of **27** or by (**b**): nucleophilic addition of 2-lithiohydroquinone dimethyl ether to **32**, and total synthesis of (±)-aureol (**4**). Cp_2_TiCl_2_ = Bis(cyclopentadienyl)titanium(IV) dichloride; DMAP = 4-(dimethylamino)pyridine; AIBN = 2,2´-azobis(2-methylpropionitrile); BF_3_·Et_2_O = boron trifluoride etherate.

Due to the fascinating chemical structures and biological properties of marine meroterpenoids, our research group and the group led by Prof. Oltra [12,30,31,32,33,34,35,36] began to develop methodologies for the total synthesis of this group of natural marine compounds. Along with Gansäuer group [37,38], we have contributed to the acquisition of marine meroterpenoids through the diastereoselective radical cyclization of epoxypolyenes catalyzed by Cp_2_TiCl as a new green monoelectronic transfer complex [39,40]. Subsequently, specifically during the synthesis of marine (±)-aureol (**4**), we reported the first example of a Lewis acid-mediated rearrangement of drimane skeleton **29** bearing an exo-olefin at C-8 to obtain a compound with the aureane skeleton **12b** (Scheme 4). Initially, our synthesis of compound **12b** with an aureane skeleton was carried out by the Cp_2_TiCl-catalyzed radical cascade cyclization of the epoxyfarnesol derivative (**27**), which was obtained from epoxyfarnesol (**24**) [12]. In this way, the mesylation of compound **24**, followed by bromination with LiBr at 0 °C and the addition of 2,5-dimethoxyphenylmagnesium bromide (**26**), furnished the epoxyfarnesol derivative **27**. Radical cascade cyclization of **27** gave the trans-fused decalin **28** possessing an exo-cyclic double bond on C-8 in a 48% yield. This cyclization catalyzed by titanocene(III) is highly diastereoselective, and four stereocenters are formed with a desired relative configuration. Furthermore, the same methodology can be used for the enantiomerically pure preparation of optically active **28** if the Sharpless asymmetric cis-hydroxylation of farnesyl acetate is used for the preparation of **24**, as described in the synthesis of β-onocerin [41]. Another advantage of titanocene(III)-catalyzed cascade cyclization of epoxyfarnesol derivative **27** is the generation of a compound with the presence of a hydroxyl group at C-3, which is present in interesting marine products such as (+)-stachyflin (**6**) (Figure 1) [42]. The deoxygenation of alcohol **28** was carried out using a two-step sequence (Scheme 4): first, the preparation of a thiocarbonate and later, its treatment with *n*-Bu_3_SnH and AIBN, leading to compound **29** in an 86% yield (two steps). Inspired by the biosynthetic hypothesis, we proposed that the treatment of **29** with BF_3_·Et_2_O could originate a rearrangement sequence of 1,2-hydride and methyl shifts. The reaction gave the desired tetrasubstituted alkene **12b** with an aureane skeleton in 63% yield, together with the undesired tetracyclic compound **30** in 30% yield. Later, Li and co-workers [14] optimized this rearrangement starting from the same drimane skeleton but with an alcohol group at the C-8 position, noting that the undesired compound **30** could be inhibited at low temperatures to improve the yield of compound **12b**. With **12b** in our hands, we completed the synthesis of marine (±)-aureol (**4**). This tetracyclic compound **4** was prepared from **12b** in three steps. The removal of the methoxy groups was achieved through a two-step oxidative (AgO, HNO_3_)/reductive (Pd/C, H_2_) sequence, which afforded the phenolic compound **31** in an 82% yield. Finally, the cyclization of hydroquinone **31**, under the same conditions reported by George and co-workers [10], gave (±)-aureol (**4**) in 62% yield.

Later, in order to consolidate the stereospecific 1,2-rearrangements as an excellent strategy towards compounds with aureane skeletons but using drimanes as starting materials, compound **29** was synthesized by the 1,2-addition of 2-lithiohydroquinone dimethyl ether to commercially available albicanal (**32**) [21]. Aldehyde **32** can also be prepared by the oxidation of albicanol (**33**), which can be obtained by the radical cyclization of epoxyfarnesyl acetate [30]. The C-C bond-forming reaction afforded a mixture of diastereomeric benzylic alcohols. Reduction of these alcohols with lithium in liquid NH_3_/THF followed by treatment with NH_4_Cl solution gave the drimane intermediate **29** in a 90% yield (two steps). Both procedures (the radical cascade cyclization of epoxyfarnesol derivatives catalyzed by titanocene(III) and the 1,2-addition of aryllithium derivatives to albicanal (**32**)) can provide compounds with a drimane skeleton with adequate functionalization of the aromatic ring to obtain highly valuable natural meroterpenoids through 1,2-rearrangements mediated by a Lewis acid as a key step (see Section 4).

### 2.3. Wu´s Synthesis of Compound ***12c***

**Scheme 5:**
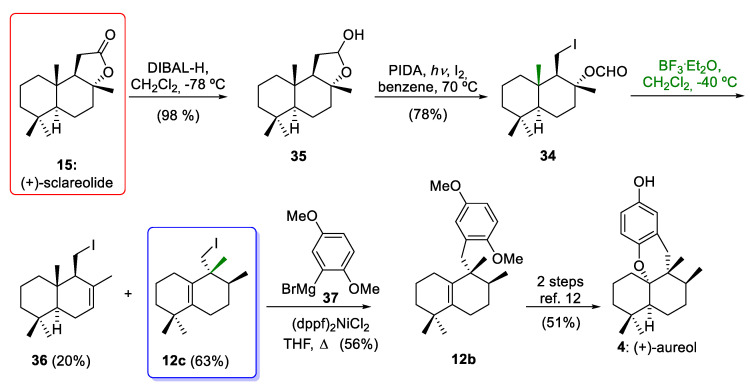
Wu´s synthesis of intermediate **12c** and formal synthesis of (±)-aureol (**4**). DIBAL-H = diisobutylaluminium hydride; PIDA = phenyliodine(III) diacetate (dppf)_2_NiCl_2_ = [1,1´-bis(diphenylphospino)ferrocene]dichloronickel(II); BF_3_·Et_2_O = borontrifluoride etherate.

In 2018, Wu and co-workers [20] reported an efficient and short synthesis of **12c** as a precursor of aureol (**4**), employing the commercially available (+)-sclareolide (**15**) as a starting material (Scheme 5). Again, the key step for the synthesis of **12c**, which has an aureane skeleton, was the stereospecific 1,2-hydride and methyl shifts of the drimane compound **34**. In this way, (+)-sclareolide (**15**) was readily converted to intermediate **34** in two steps, including the reduction of the starting material using diisobutylaluminium hydride (DIBAL-H) to give **35** and the C-C bond cleavage conditions reported by Suarez´s group (I_2_/PIDA/hν) [43] of **35** to afford **34**. These conditions led to the formation of the formate derivative of iododrimanol **34** in a 76% overall yield. When drimane **34** was treated with BF_3_·Et_2_O, the tetrasubstituted alkene **12c** and the undesired compound **36** were obtained in 63% and 20% yields. The drimane **34**, which is equipped with a versatile iodine functionality, can be employed as a common precursor of marine meroterpenoids with aureane or avarane skeletons, as it allows the incorporation of the aromatic ring either through the 1,2-addition of aryllithium derivatives or through the Negishi coupling of aryl derivatives, as will be discussed in Section 4. With tetrasubstituted alkene **12c** and an aryl Grignard reagent **37,** the tricyclic compound **12b** was prepared, which is an advanced intermediate in the synthesis of aureol (**4**), as reported by our research group [12].

### 2.4. Li’s Synthesis of Compound ***12b***

**Scheme 6:**
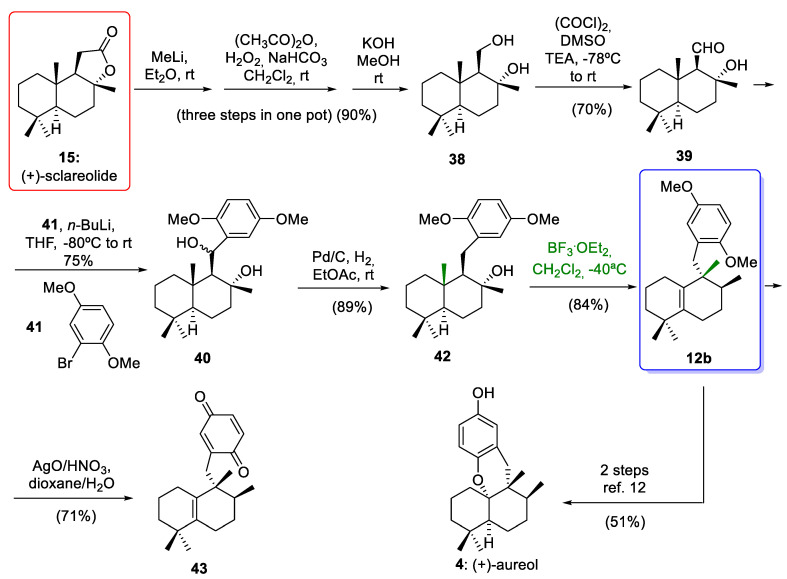
Preparation of compound **12b** via stereospecific 1,2-rearrangements and formal synthesis of (+)-aureol (**4**). (CH_3_CO)_2_O = acetic anhydride; (COCl)_2_ = oxalyl chloride; BuLi = butyllithium; TEA = triethanolamine; BF_3_·Et_2_O = boron trifluoride etherate.

Recently, Li and co-workers [14] reported the rearrangement of a drimane skeleton to an aureane skeleton employing as starting material commercially available (+)-sclareolide (**15**) (Scheme 6). In addition, for the first time, this research group included a promising study that provides new and hopeful ideas for the synthesis of compounds with an avarane skeleton from compounds with an aureane skeleton. This study provides a new pathway for the synthesis of avarane compounds using cyclization mediated by a Lewis acid.

(+)-Sclareolide (**15**) was converted to diol **38** using a one-pot, three-step sequence in 90% yield over three steps. The oxidation of diol **38** gave aldehyde **39** in a 70% yield. The cross-coupling reaction between an aryllithium derivative of **41** and aldehyde **39** led to diastereomeric benzyl alcohol **40** in 75% yield. Deoxygenation of benzyl alcohol **40** by hydrogenolysis afforded tertiary alcohol **42,** which led to the aureane compound **12b** upon treatment with BF_3_·Et_2_O through a stereospecific rearrangement of 1,2-hydride and methyl shifts. This key step gave the desired compound **12b** with an aureane skeleton in a moderate yield. This moderate yield was obtained when the rearrangement was carried out at −50 °C and low reaction times. Optimization of the reaction conditions, using the same Lewis acid at −40 °C and 11 h of reaction, afforded the compound **12b** in a higher yield (84%). As the tetrasubstituted olefin **12b** is an intermediate in the total synthesis of aureol (**4**) reported by Rosales Martínez and co-workers [12], this work constitutes a formal synthesis of this interesting marine natural product (Scheme 6).

**Scheme 7:**
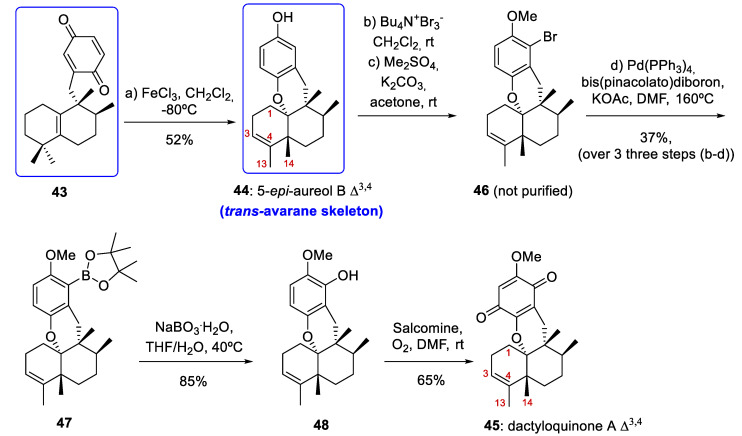
Preparation of compound **44** with an avarane skeleton and synthesis of dactyloquinone A Δ^3,4^ (**45**). DMF = dimethylformamide; salcomine = *N,N*´-bis(salicylidene)ethylenediaminocobalt(II).

As we have already mentioned, the most notable milestone in the work published by Li and co-workers [14] was the preparation of tetracyclic meroterpenoids with an avarane skeleton. These compounds were obtained by a bioinspired Lewis acid-catalyzed cyclization reaction of quinone **43** (prepared from compound **12b**) with the concomitant migration of C-4 Me-groups through 1,2-rearrangement leading to compounds with an avarane skeleton (Scheme 7).

Therefore, in this study, the authors simultaneously described the preparation of compounds with aureane (compound **12b**, Scheme 6) and avarane (compound **44**, Scheme 7) skeletons. This finding supports the affirmation that the bioinspired sequence of 1,2-hydride and methyl shifts in compounds with a drimane skeleton is a general strategy that unifies the synthesis of a large group of marine meroterpenoids with aureane and avarane skeletons, starting from commercially available compounds with drimane skeletons.

In this way, when tetrasubstituted olefin **12b** was treated with AgO/HNO_3_, quinone **43** was obtained in a yield of 71% (Scheme 6). Several Lewis acids were tested for the cascade reaction that allows the cyclization/rearrangement of the quinone **43** into **44**. After optimization, treatment of **43** in a gram scale (2.0 g) with FeCl_3_ at −80 °C afforded the trans-avarane compound **44** in 52% yield with satisfactory stereoselectivity (Scheme 7). This trans-avarane compound **44** was used as starting material for the synthesis of non-natural dactyloquinone A Δ^3,4^ (**45**) (Scheme 7). From **44**, the sequence was a selective bromination followed by methylation, a Miyaura borylation, and two final oxidations with NaBO_3_ and O_2_/salcomine. As a result, dactyloquinone A Δ^3,4^ (**45**) was obtained in a 20% overall yield from **46** (Scheme 7).

**Scheme 8:**
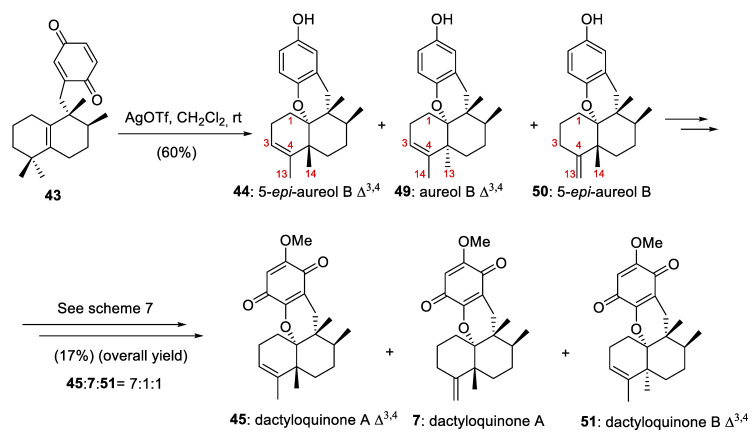
Preparation of of 5-*epi*-aureol B Δ^3,4^ (**44**), aureol B Δ^3,4^ (**49**), 5-*epi*-aureol B (**50**), dactyloquinone A Δ^3,4^ (**45**), dactyloquinone A (**7**) and dactyloquinone B Δ^3,4^ (**51**).

On the other hand, the reaction of the quinone **43** at the gram scale (1.5 g) with AgOTf at room temperature in CH_2_Cl_2_ afforded an inseparable mixture of 5-*epi*-aureol B Δ^3,4^ (**44**), aureol B Δ^3,4^ (**49**), and 5-*epi*-aureol B (**50**) in a 60% combined yield (Scheme 8). When this mixture was treated under the conditions reported in Scheme 7, dactyloquinone A Δ^3,4^ (**45**), dactyloquinone A (**7**), and dactyloquinone B Δ^3,4^ (**51**) (7:1:1) were obtained with a total yield of 17% (Scheme 8). Compounds **7** and **45** proved to be inseparable by HPLC purification.

Dactyloquinone A (**7**) is a marine sesquiterpenoid quinone isolated from marine sponges [44] in very small amounts, limiting the study of its biological properties.

## 3. Probable Reaction Mechanism for BF_3_·Et_2_O Mediated 1,2-Hydride and Methyl Shifts to Generate Aureane Compounds 12a–d

**Scheme 9:**
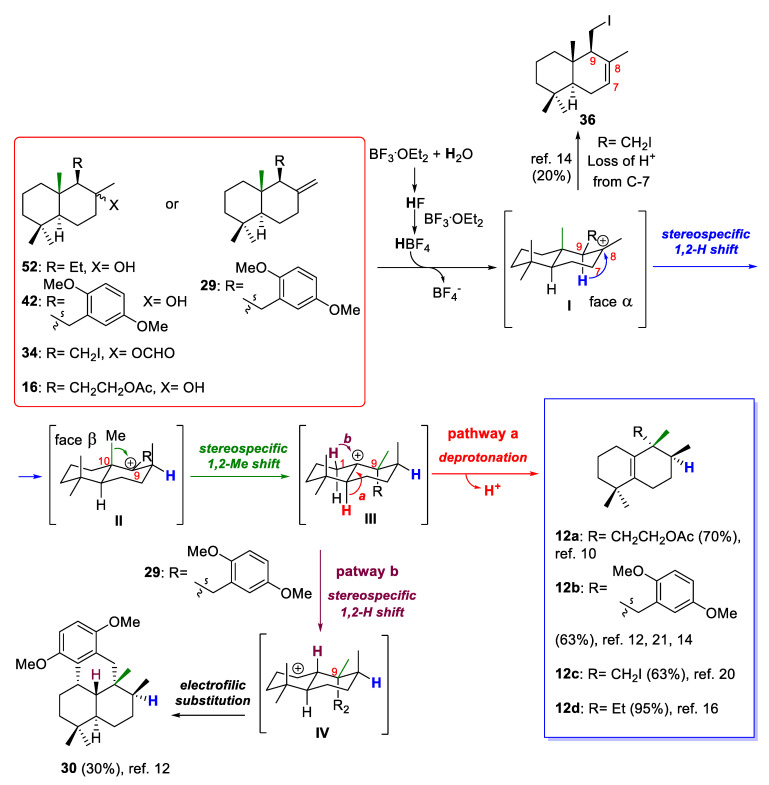
Probable reaction mechanism for BF_3_·Et_2_O-promoted 1,2-hydride and methyl shifts from compounds with drimane skeletons **16**, **29**, **34**, **42** and **52**.

Although mechanistic studies of the rearrangements of drimanes via sequential 1,2-hydride and methyl shifts have not been performed in detail [21], the remarkable stereocontrolled rearrangements of drimanes **16**, **29**, **34**, and **52** can be rationalized by the mechanistic route shown in Scheme 9, which is based on the proposed mechanism for similar acid-catalyzed rearrangements [45,46]. From drimanes **16**, **29**, **34**, and **52,** the reaction process would involve four possible carbocationic intermediates **I**–**IV**. The cationic rearrangements would initially involve a proton transfer since it is known that pure Lewis acids are not ideal initiators in alkene cationic polymerization [47]. Additionally, it is known that BF_3_·Et_2_O is a very moisture-sensitive Lewis acid, and over time the HF that generates from the hydrolysis of BF_3_ will react with an excess of this Lewis acid to produce HBF_4_, which is a strong acid, and possibly trigger the cationic rearrangement. Thus, when the hydroxyl group of the drimanes (**16**, **52**), the formate group of drimane **34**, or the exocyclic double bond of drimane **29** is activated by a proton, the tertiary carbocation **I** is generated, which then would furnish the carbocationic intermediate **II** by migration of the C-9 hydrogen to the C-8 carbocation center. From a stereochemical point of view, the configuration of C-9 allows a 1,2-hydrogen shift on the α-face of the intermediate **I** to generate the intermediate **II** or trisubstituted alkene **36** after losing a proton at C-7 (this undesirable product was obtained when iododrimanol formate **34** was used as starting material in the rearrangement reaction). Carbocationic intermediate **II** would undergo a 1,2-methyl shift from the methyl group C-10 to the carbocationic center C-9 within the β-face of the molecule to furnish the carbocationic intermediate **III**, in which the loss of a proton at C-5 leads to the desirable compounds **12a**–**d** (pathway a, Scheme 9). 

On the other hand, it has been observed that when the C-11 of the starting material was linked to an aromatic derivative ring (**29**), the carbocationic intermediate **III** underwent a 1,2-hydride shift from the C-1 position to give the intermediate **IV** (pathway b, Scheme 9), the carbocation of which was trapped by the aromatic ring by electrophilic substitution to afford the undesirable minor tetracyclic compound **30**. In both pathways, a proton is liberated, which can react with a more non-activated hydroxyl group or exocyclic alkene in the starting drimanes **16**, **29**, **34**, and **52** to follow the catalytic cycle.

The simultaneous generation of tetrasubstituted alkene **12a**–**d** and sub-products **30** and **36** suggests that these 1,2-rearrangements are not part of a concerted reaction. These rearrangements would proceed via an unconcerted mechanism involving a series of rapidly interconverting carbocationic intermediates. The major products with aureane skeletons (**12a**–**d**) are shown in Scheme 9, together with the best yields obtained after optimization of the 1,2-rearrangement reactions of the different starting drimanes (see the references shown in Scheme 9). The optimization of this rearrangement towards the formation of compounds with aureane skeletons is the result of many factors, which mainly include Lewis acid equivalents, temperature and reaction time [14]. The non-optimization of the rearrangement towards aureane skeletons generates mainly drimanes with a trans-decalin structure and a *tetra* (Δ^8^) or *tri*-substituted double bond (Δ^7^) [16].

## 4. Future Perspectives

Although we have successfully discussed different synthetic routes for marine meroterpenoids with aureane or avarane skeletons from available drimanes, with the key step being a cascade of 1,2-rearrangements, the alternative synthesis of other marine compounds with similar structures is desirable to illustrate the flexibility of this rearrangement as a key step. The precursor drimanes of compounds with aureane or avarane skeletons can be prepared by a convergent synthetic route, where the C-C bond between the drimane skeleton and the aromatic ring is formed either through a Negishi coupling or the 1,2-addition of an arylmetal derivative, or, alternatively, by a linear synthetic route using as starting material epoxipolyene derivatives. The synthetic value of the stereospecific 1,2-hydride and methyl shifts is demonstrated by the reported or future synthesis of select members of marine meroterpenoids in a concise and practical manner.

**Scheme 10:**
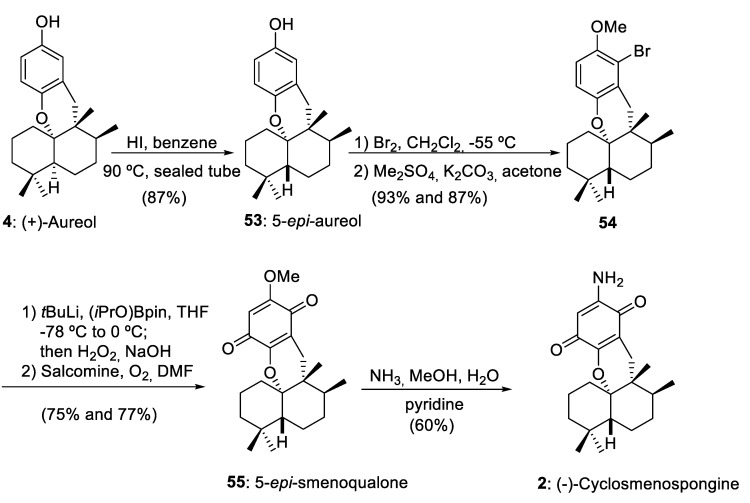
Preparation of 5-*epi*-smenoqualone (**55**) and (−)-cyclosmenospongine (**2**) from aureol (**4**). Salcomine = *N,N*´-bis(salicylidene)ethylenediaminocobalt(II). (*i*PrO)Bpin = isopropoxy pinacolborane.

As we have commented in Section 2, using as starting material drimanes **16**, **29,** and **34** synthesized by a convergent or linear route, marine aureol (**4**) has been obtained efficiently using as a key step a 1,2-rearrangement mediated by BF_3_·Et_2_O. The important epimerization of aureol (**4**) into 5-*epi*-aureol (**53**) reported by Magauer and co-workers [15] opened the door to the preparation of other marine aureane skeletons with a trans-decalin structure, such as 5-*epi*-smenoqualone (**55**) and (−)-cyclosmenospongine (**2**) (Scheme 10). 

The sequence including the epimerization of (+)-aureol (**4**), selective bromination and subsequent methylation, boronation-oxidation, and final aminolysis furnished (−)-cyclomenospongine (**2**) with a 24% overall yield from aureol (**4**). Tetracyclic meroterpenoid (−)-cyclomenospongine (**2**) is a natural compound isolated from a marine source [48] and with remarkable biological activity [15]. Magauer and co-workers have realized an important contribution to the synthesis of this compound using a highly modular synthetic strategy [15,49,50].

In addition to the synthesis of (+)-aureol (**4**) and (−)-cyclosmenospongine (**2**), other marine meroterpenoids with aureane skeletons can be easily prepared using the 1,2-rearrangements widely discussed in this article as a key step. In this way, the future syntheses of (+)-stachyflin (**6**), (+)-neomamanuthaquinone (**1**), and (+)-stronglyn A (**5**) are described below.

**Scheme 11:**
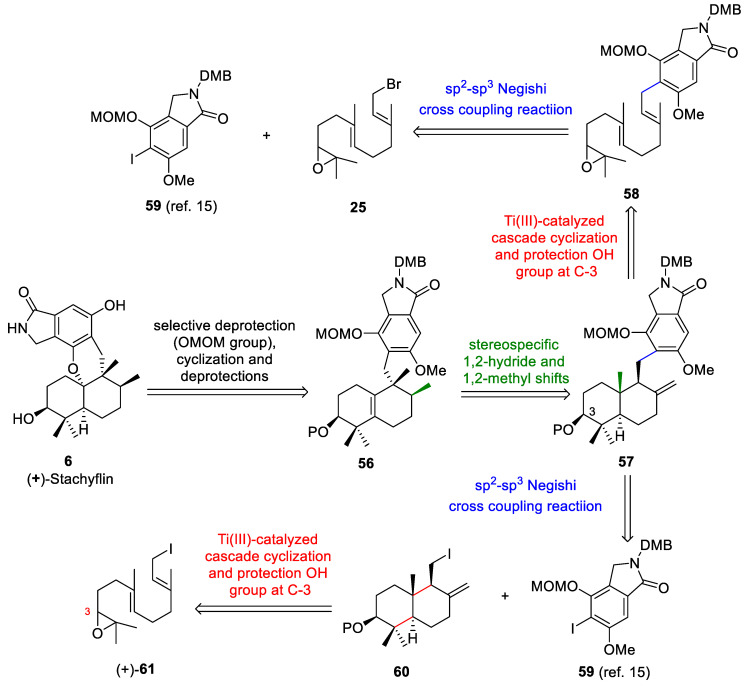
Retrosynthetic analysis of (+)-stachyflin (**6**).

The retrosynthetic analysis of (+)-stachyflin (**6**) (Scheme 11) is based on: (a) the acid-mediated cyclization of the intermediate obtained from **56** after selective deprotection using Magauer´s conditions [15], (b) the key rearrangement reaction of **57** to give **56** using BF_3_·Et_2_O, (c) Cp_2_TiCl-catalyzed cascade cyclization of the epoxyfarnesol derivative **58** and protection of the OH group at C-3 to give **57**, and (d) the cross-coupling reaction between the bromide **25** and isoindoline derivative **59** to afford **58**. Alternatively, compound **57** could be obtained through a coupling reaction between drimane **60** and the isoindoline derivative **59**. The trans-decalin **60** could be obtained through the titanocene(III)-catalyzed cascade cyclization of the epoxyfarnesol derivative **61**.

(+)-Stachyflin (**6**) is a marine meroterpenoid with a fascinating chemical structure and biological activities [15]. It has been published that the anti-influenza A virus activity of this compound is 250 times higher than that of zanamivir (IC_50_ = 0.75 mM) and 1760 times higher than that of amantadine (IC_50_ = 5.3 mM) [42,51]. This compound was isolated from a marine fungus by the Shionogi research group [52]. The total synthesis of this compound has been reported by several research groups [5,7,9,15].

**Scheme 12:**
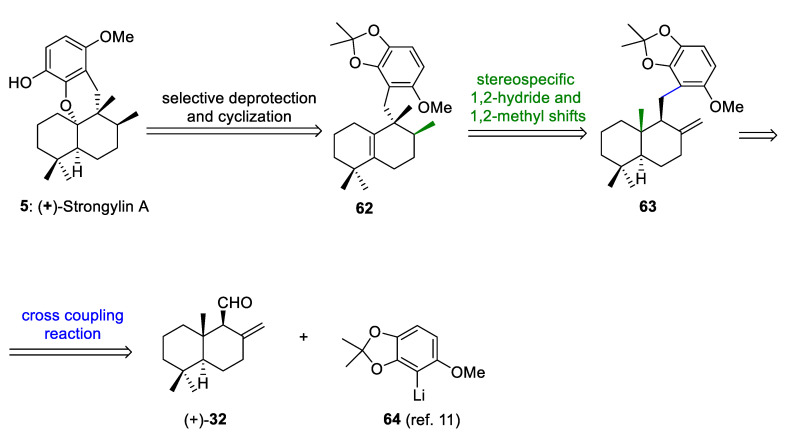
Retrosynthetic analysis of (+)-strongylin A (**5**).

The retrosynthetic analysis of (+)-strongylin A (**5**) is outlined in Scheme 12. This retrosynthetic sequence is based on: (a) the selective deprotection and biomimetic acid-mediated cyclization of the hydroquinone of **62** to form **5**, (b) a biosynthetically inspired sequence of 1,2-hydride and methyl shifts of **63** to form **62**, and (c) a cross-coupling reaction between abicanal **32** and aryllithium **64** to afford a mixture of benzyl alcohols, which after removal of the free hydroxy groups would give **63**. Albicanal (**32**) can be synthesized by the oxidation of albicanol (**33**), which is easily prepared through the Cp_2_TiCl-catalyzed radical cyclization of epoxyfarnesyl acetate, as previously reported by us and others [21,30,53].

(+)-Strongylin A (**5**) has been isolated from marine sponges [54,55] and possesses interesting biological activities [15,54]. The first total synthesis of (+)-strongylin A was reported by Katoh and co-workers [11] and later by Magauer and co-workers [15] through a modular synthetic strategy.

**Scheme 13:**
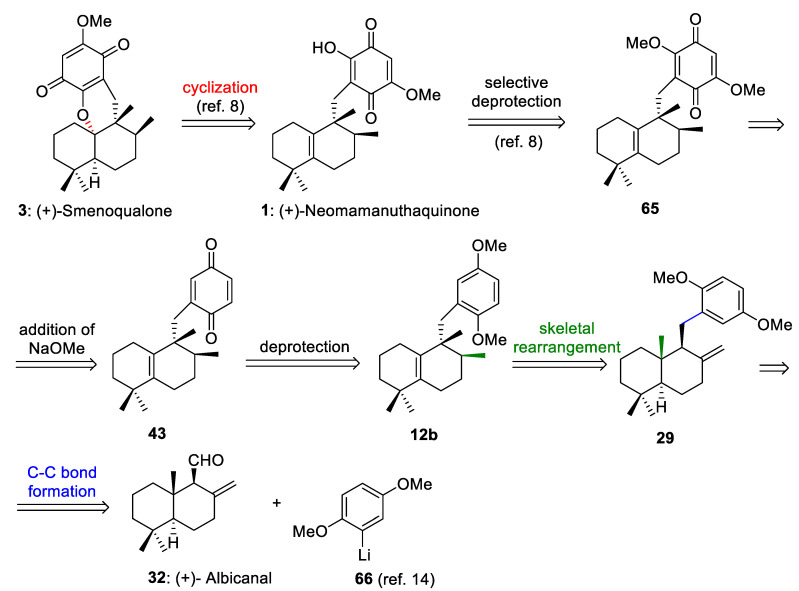
Retrosynthetic analysis of (+)-neomamanuthaquinone (**1**) and (+)-smenoqualone (**3**).

The retrosynthetic analysis of (+)-neomamanuthaquinone (**1**) and (+)-smenoqualone (**3**) is shown in Scheme 13. This retrosynthetic route is based on: (a) the selective deprotection of the methoxy groups of **65** to generate **1**, (b) the addition of NaOMe to the quinone ring of **43** to afford **65**, (c) the deprotection of both methoxy groups in **12b** to produce **43**, (d) stereospecific 1,2-hydride and methyl shifts to transform **29** into **12b**, and (e) a C-C bond formation reaction between albicanal (**32**) and 2-lithiohydroquinonedimethylether (**66**). As neomamanuthaquinone (**1**) is the preliminary intermediate in Marcos´s synthesis of (−)-smenoqualone [8], the retrosynthetic analysis shown in Scheme 13 constitutes a formal synthesis of (+)-smenoquealone (**3**) (Scheme 13).

(+)-Neomamanuthaquinone (**1**) is a marine quinone isolate from marine sponges [56], which shows cytotoxic [56], hemolytic [57] and antioxidant activities [58]. The enantiomer of this interesting marine natural compound was synthesized by Marcos et al. [8].

(+)-Smenoquealone (**3**) is another tetracyclic meroterpenoid with biological activity isolated from a marine sponge [59]. This compound has been synthesized by several research groups [8,13,15].

## 5. Conclusions

In summary, we have established a synthetic strategy for the preparation of a large number of marine natural products with aureane and avarane skeletons. The key step of this synthetic strategy is a biosynthetically inspired rearrangement of 1,2-hydride and methyl shifts from available drimanes obtained from (+)-sclareolide (**15**), albicanal (**32**), and epoxy farnesol (**24**). This article reviews the total synthesis of aureol (**4**), (−)-cyclomenospongine (**2**), and (−)-dactyloquinone A (**7**) and proposes future syntheses for (+)-neomamanuthaquinone.(**1**) (+)-smenoqualone (**3**), (+)-strongylin A (**5**), and (+)-stachyflin (**6**) using the aforementioned rearrangement as a key step. Consequently, this general synthetic strategy demonstrates its versatility and power for the construction of natural marine compounds with aureane and avarane skeletons that could be used to address relevant biological issues.

## Data Availability

Not applicable.
